# Metabolic dysfunction-associated steatotic liver disease in chronic hepatitis B patients: risks of severe liver disease and cardiovascular disease

**DOI:** 10.3389/fcimb.2026.1806927

**Published:** 2026-06-09

**Authors:** Ningxin Zhang, Zhongwei Xu, Fei Lin, Haozhi Fan, Dehong Zhou, Shiqiu Zheng, Peng Jin, Huiquan Gu, Chengxiao Yu, Longfeng Jiang

**Affiliations:** 1 Department of Infectious Diseases, The First Affiliated Hospital with Nanjing Medical University, Nanjing, Jiangsu, China; 2 Health Management Center, The First Affiliated Hospital with Nanjing Medical University, Nanjing, Jiangsu, China; 3 Department of Information Management, The First Affiliated Hospital with Nanjing Medical University, Nanjing, China; 4 Jiangsu Clinical Medicine Research Institute, Nanjing, China; 5 Department of Health Management, School of Public Health, Nanjing Medical University, Nanjing, Jiangsu, China

**Keywords:** cirrhosis, coronary heart disease, HBsAg, hepatocellular carcinoma, severe liver disease

## Abstract

**Objective:**

To assess the association between comorbid Metabolic Dysfunction-Associated Steatotic Liver Disease (MASLD) and the risk of developing severe liver disease and cardiovascular disease (CVD) in patients with Chronic Hepatitis B (CHB).

**Methods:**

CHB Patients were continuously recruited from 2012 to 2022 using the electronic medical record system, and divided into CHB-no MASLD and CHB-MASLD groups. Follow-up continued until January 2024, aiming to compare differences of cumulative incidence between the two groups and to analyze associated risk factors.

**Results:**

8,052 patients were included with a median follow-up of 3.9 years. Compared with CHB-no MASLD group, MASLD was independently associated with a 39% lower risk of severe liver disease (adjusted hazard ratio [aHR]=0.61, 95% confidence interval [CI] 0.49-0.76, *p* < 0.001), with similar effects in cirrhosis (aHR=0.61, 95% CI 0.45-0.82, *p=*0.001) and hepatocellular carcinoma (HCC) (aHR=0.57, 95% CI 0.41-0.80, *p* = 0.001). Stratified analyses indicated the protection effect was more significant in the HBsAg-positive group. Conversely, MASLD was associated with higher CVD risk (aHR=1.16, 95% CI 0.96-1.41, *p=*0.130), driven by coronary artery disease (aHR=1.91, 95% CI 1.34-2.74, *p* < 0.001) and arrhythmia (aHR=1.44, 95% CI 1.00-2.08, *p=*0.053). These findings remained consistent after propensity score matching (PSM).

**Conclusion:**

MASLD may reduce the risk of severe liver disease through certain mechanisms, yet it also constitutes a risk factor for CVD, particularly among HBsAg-positive patients.

## Introduction

Hepatitis B virus (HBV) is a hepatotropic virus that may cause persistent infection, ultimately leading to cirrhosis and hepatocellular carcinoma (Iannacone and Guidotti, 2022; Global prevalence, cascade of care, and prophylaxis coverage of hepatitis B in 2022: a modelling study, 2023). Although hepatitis B vaccination has suppressed the transmission of HBV, China is still facing a huge burden of hepatitis B. According to a recent statistic, approximately 75 million people in China have hepatitis B, accounting for nearly one-third of the 254 million people chronically infected with hepatitis B worldwide (Lou et al., 2024).

And over the past two decades, the prevalence and incidence of fatty liver (FL) have been increasing globally due to energy-dense but nutrient-poor diets, sedentary lifestyles and physical inactivity, and an aging population. In China, the prevalence of FL is about 44.39% and has been on the rise in recent years, having replaced chronic viral hepatitis as the number one chronic liver disease (Man et al., 2023). Accordingly, among HB patients, the number of patients with combined FL is increasing, and the prevalence rate is estimated to be more than 30% (Jiang et al., 2021; Zheng et al., 2021). The prognosis of this group of patients is a matter of concern. However, controversy still exists regarding their combined clinical impact. Li et al. found that the 10-year cumulative incidence of cirrhosis and HCC was lower in CHB patients with FL compared with no-FL CHB (Li et al., 2021). Wong et al. conducted a meta-analysis of 19 studies enrolling 10,301 patients found that all outcomes evaluated including HCC, cirrhosis, mortality, and HBsAg serologic clearance favored CHB patients with comorbid FL. The protective effect of FL was stable in subgroup analyses stratified by age, sex, baseline cirrhosis, diabetes, and HBeAg (Wong et al., 2023). In contrast, studies based on liver biopsy cohorts reported a higher risk of HCC in CHB patients with FL compared to CHB without MASLD (van Kleef et al., 2021; Patmore et al., 2023).

On the other hand, MAFLD also places patients at higher cardiovascular risk, with higher severity of FL being associated with higher risk of fatal and nonfatal CVD events, including left ventricular dysfunction, cardiac arrhythmias, and ischemic stroke (Duell et al., 2022). A large retrospective cohort study from Taiwan reported a cardiovascular-related mortality rate of 11.4% in the CHB combined MAFLD group, which was higher than that of 8.9% in the uncomplicated MAFLD group (Huang et al., 2024a). However, there is still a lack of reports on the incidence of CVD in the population with CHB combined with MASLD.

Considering the gradually increasing prevalence of steatotic liver disease in CHB patients (Li et al., 2025), and the unspecified impact of CHB combined with MASLD on the incidence of severe liver disease and CVD, this study investigated the clinical characteristics in CHB patients who presented to our hospital in recent years, and compared the differences in clinical outcomes between CHB-MASLD and CHB-no MASLD group.

## Methods

### Study design

A single-center retrospective cohort study utilized the electronic medical record system of Jiangsu Province Hospital. CHB patients who underwent abdominal imaging (ultrasound or CT) between 2012–2023 were screened. Participants were followed until 1 January 2024 or development of primary outcomes. The current study protocol was approved by the Ethics Review Committee of Jiangsu Province Hospital under Ethics No. 2025-SR-110. All authors had access to the study data and approved the submitted manuscript.

### Study population

From January 2012 to January 2024, 31,373 CHB patients were screened from the Clinical Disease-Specific Database of Jiangsu Province Hospital (CDSD). The inclusion criteria for the study participants were as follows: (1) patients diagnosed with CHB at Jiangsu Provincial People’s Hospital with at least 2 visits; (2) availability of liver imaging examinations performed between January 2012 and December 2022. The exclusion criteria included: (1) a clearly documented history of excessive alcohol consumption (>30g/day men, >20g/day women); (2) diagnoses of cardiovascular disease, severe liver disease, and malignancy prior to the time of enrollment and within 1 month after enrollment; (3) incomplete clinical data. Finally 8,052 patients comprised the analytic cohort for this follow-up investigation of severe liver disease and CVD, and propensity score matching was performed, resulting in a final matched cohort of 2,958 individuals. (**Supplementary Figure S1**).

### Definition of diseases

Diagnostic criteria for Metabolic dysfunction-associated steatotic liver disease (MASLD): (1) Imaging findings consistent with hepatic steatosis; (2) Presence of at least one cardiometabolic risk factor: ①Overweight: Body mass index (BMI) ≥23 kg/m²; ②Type 2 diabetes; ③Hypertension; ④Elevated plasma triglycerides: Fasting serum triglycerides ≥1.7 mmol/L (≥150 mg/dL); ⑤Reduced plasma high-density lipoprotein cholesterol (HDL-C): <1.0 mmol/L (40 mg/dL) in men or <1.3 mmol/L (50 mg/dL) in women.

CHB was defined as a diagnosis of chronic hepatitis B virus infection confirmed based on the patient’s symptoms, medical history, and auxiliary examinations, or possessing diagnostic code B 18 according to the International Classification of Diseases codes (ICD-10 codes WHO version). Hypertension/diabetes was defined as a confirmed diagnosis or current use of antihypertensive/antidiabetic medication. Antiviral therapy is defined as a prescription for nucleoside/nucleotide analogues (NAs).

### Definition of clinical outcomes

The clinical outcomes of the patients were collected according to the ICD-10 codes. The severe liver diseases discussed in this study included cirrhosis (K74.0/2/6), HCC (C22.0), liver failure (K72.0/1/9). The CVD included coronary disease (I20-I25), arrhythmia (I47-I49), heart failure (I50) and stroke (I60-I63).

### Key variables for adjustment and subgroup analysis

Socioeconomic factors, including age, sex, health screening results, including total cholesterol (TC), high-density lipoprotein cholesterol (HDL), low-density lipoprotein cholesterol (LDL), triglycerides (TG), alanine aminotransferase (ALT), aspartate aminotransferase (AST), platelet count (PLT), fasting glucose (GLU), HBV DNA titer, hepatitis B surface antigen (HBsAg), hepatitis B e antigen (HBeAg) and history of chronic medical conditions including hypertension, diabetes mellitus and antiviral therapy were included in the statistics and adjusted. All blood specimens were collected at the same time as the imaging tests were performed.

### Statistical methods

The continuous variables were expressed by and median ± Standard Deviation (SD) and compared by t-test. The categorical data were expressed in numbers (percentage) and compared by Chi-square test. The cumulative incidence was calculated by the Kaplan-Meier method. Adjusted hazard ratios (aHR) with 95% CIs were derived from multivariable Cox proportional hazards models adjusted for baseline characteristics. Cumulative incidence by MASLD subgroups was visualized, and plotted by Graphpad Prism.

Propensity score matching (PSM) was performed based on baseline age, sex, body mass index (BMI), hypertension, diabetes, HBsAg status, HBV DNA level, and FIB-4 score. Matching was carried out at a 1:3 ratio with a caliper width set within 0.20 standard deviations of the logit of the propensity score. In the well-balanced cohort, univariate and multivariate Cox proportional hazards regression models were employed to assess the association between MASLD and the development of severe liver events and cardiovascular disease (CVD). Analyses used IBM SPSS 25.0 and R 4.3.1, with statistical significance defined as *p* < 0.05.

## Result

### Baseline characteristics of the study population

This cohort enrolled 8,052 subjects, with 1,609 (19.98%) in CHB-MASLD group and 6,443 (80.02%) in CHB-no MASLD group. Significant differences existed in demographics and metabolic profiles. CHB-MASLD group had lower mean age (47.04 ± 11.43 vs 49.00 ± 12.83, *p* < 0.001), higher male proportion (71.60% vs 58.25%, *p* < 0.001), elevated rates of hypertension (29.65% vs 21.53%, *p* < 0.001) and diabetes (19.02% vs 12.18%, *p* < 0.001), and fasting glucose levels (5.74 ± 2.12 vs 5.35 ± 1.90, *p* = 0.006).

Lipid profiles indicated significant dysregulation in CHB-MASLD group. Compared to the no-MASLD group, it exhibited higher TC (1.65 ± 1.11 vs 1.21 ± 0.77, *p* < 0.001), TG (4.60 ± 1.14 vs 4.35 ± 1.21, *p* = 0.017), and LDL (2.91 ± 0.81 vs 2.67 ± 0.84, *p* < 0.001), but lower HDL (1.10 ± 0.31 vs 1.18 ± 0.39, *p* < 0.001). Although AST and ALT did not differ significantly, non-invasive fibrosis indices FIB-4 suggested lower risk in CHB-MASLD group (2.04 ± 5.67 vs 3.06 ± 8.31, *p* < 0.001). At the virological level, the proportion of patients with low-level HBV DNA was higher in the MASLD group (*p* = 0.003), while the positive rate of HBsAg and HBeAg lacked clinical significance (**Table 1**).

**Table 1:** Baseline clinical characteristics.

Characteristic	Total	No-MASLD group	MASLD group	*P*
Age (years)	8052	49.00 ± 12.83	47.04 ± 11.43	**<0.001**
Male sex	8052	3753 (58.25%)	1152 (71.60%)	**<0.001**
Hypertension (mmHg)	8052	1387 (21.53%)	477 (29.65%)	**<0.001**
Diabetes Mellitus	8052	785 (12.18%)	306 (19.02%)	**<0.001**
BMI (kg/m^2^)	8052	25.20 ± 3.62	26.46 ± 4.28	**<0.001**
GLU (mmol/L)	7762	6.08 ± 2.10	6.33 ± 1.64	**<0.001**
TC (mmol/L)	8052	1.21 ± 0.77	1.65 ± 1.11	**<0.001**
TG (mmol/L)	8052	4.35 ± 1.21	4.60 ± 1.14	**<0.001**
HDL (mmol/L)	7709	1.18 ± 0.39	1.10 ± 0.31	**<0.001**
LDL (mmol/L)	7709	2.67 ± 0.84	2.91 ± 0.81	**<0.001**
ALT (U/L)	8052	70.19 ± 211.32	86.71 ± 293.76	**0.034**
AST (U/L)	8052	57.18 ± 179.15	59.38 ± 246.72	0.686
PLT (10^9^/L)	4688	173.60 ± 76.10	188.93 ± 70.96	**<0.001**
FIB-4	8051	3.06 ± 8.31	2.04 ± 5.67	**<0.001**
HBV DNA	6377	5037 (78.99%)	1340 (21.01%)	**0.008**
<2*10^3^IU/mL	4539	3542 (70.32%)	997 (74.40%)	
2*10^3^-2*10^6^IU/mL	1287	1049 (20.83%)	238 (39.50%)	
≥2*10^6^IU/mL	514	420 (8.34%)	94 (7.01%)	
HBsAg positive	3966	2827 (85.7%)	556 (83.5%)	0.147
HBeAg positive	3966	564 (17.1%)	102 (15.4%)	0.288

BMI, body mass index; GLU, fasting glucose; TC, total cholesterol; HDL, high-density lipoprotein cholesterol; LDL, low-density lipoprotein cholesterol; TG, triglycerides; ALT, alanine aminotransferase; AST, aspartate aminotransferase; PLT, platelet count; FIB-4, Fibrosis-4 Index; HBsAg, Hepatitis B surface antigen; HBeAb, Hepatitis B e antigen. *P*-values<0.05 are shown in bold.

### Uni-variate and multi-variate analysis of MASLD and clinical outcomes

#### Severe liver disease risk

**Figure 1:**
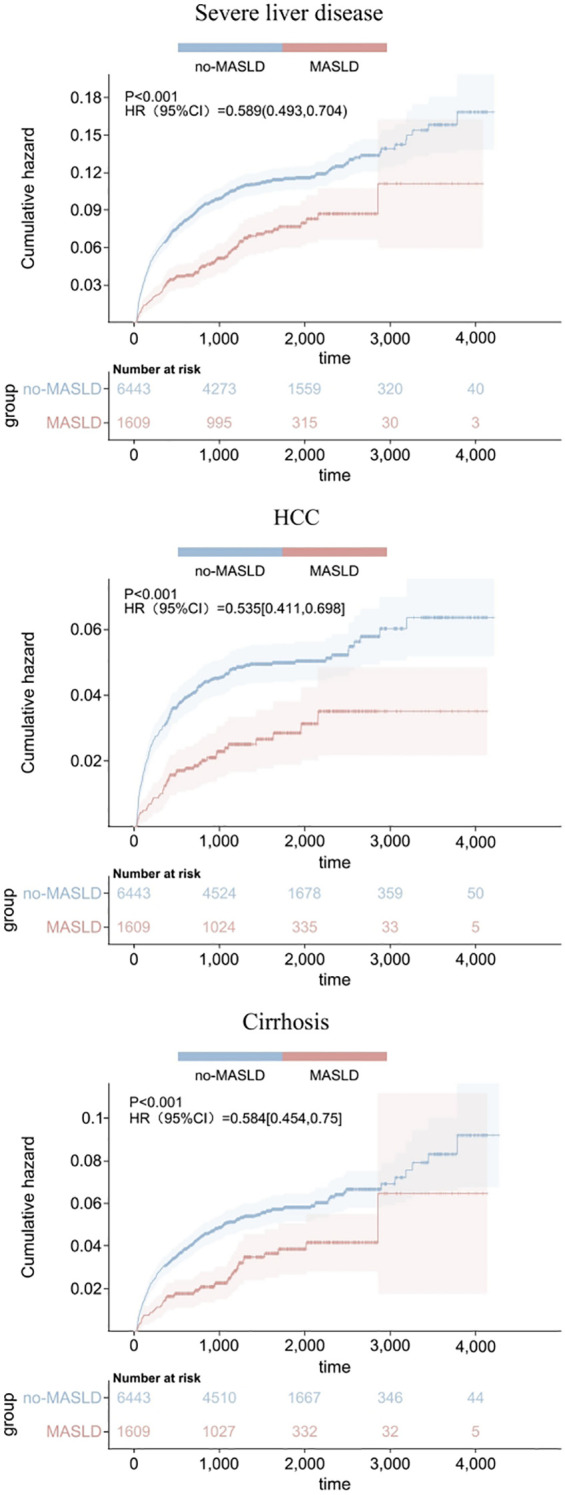
Association between comorbid MASLD and the occurrence of severe liver disease.

Follow-up of 31,806 person-years revealed a total of 764 cases, with an incidence density of approximately 24.02/1000 person-years. Univariate analysis showed MASLD reduced severe liver disease risk (HR 0.59, 95% CI 0.48-0.73, *p* < 0.001), with consistent protection against HCC (HR 0.54, 95% CI 0.39-0.74, *p* < 0.001) and cirrhosis (HR 0.54, 95% CI 0.43-0.79, *p* < 0.001)(**Figure 1**). After adjusted for sex, age, BMI, hypertension, diabetes at baseline, MASLD remained protective effect (severe liver disease: aHR 0.61, 95% CI 0.49-0.76, *p* < 0.001; HCC: aHR 0.57, 95% CI 0.41-0.80, *p* = 0.001; cirrhosis: aHR 0.61, 95% CI 0.45-0.82, *p=*0.001) (**Table 2**).

**Table 2:** The relationship between MASLD and clinical outcomes in overall cohort.

Characteristic	No. of participants	No. of cases	aHR (95.0%CI)	*P*
Severe liver disease	8052	764	**0.610 (0.490-0.758)**	**<0.001**
HCC	8052	344	**0.574 (0.410-0.804)**	**0.001**
Cirrhosis	8052	389	**0.606 (0.445-0.824)**	**0.001**
Liver failure	8052	31	1.394 (0.624-3.111)	0.418
Cardiovascular disease	8052	594	1.160 (0.957-1.406)	0.130
Coronary heart disease	8052	145	**1.913 (1.338-2.736)**	**<0.001**
Arrhythmia	8052	150	1.438 (0.996-2.076)	0.053
Heart failure	8052	59	0.821 (0.406-1.661)	0.584
Cerebrovascular events	8052	378	1.037 (0.813-1.323)	0.768

Adjusted for sex, age, BMI, hypertension and diabetes at baseline. *P*-values<0.05 are shown in bold.

#### Cardiovascular disease risk

**Figure 2:**
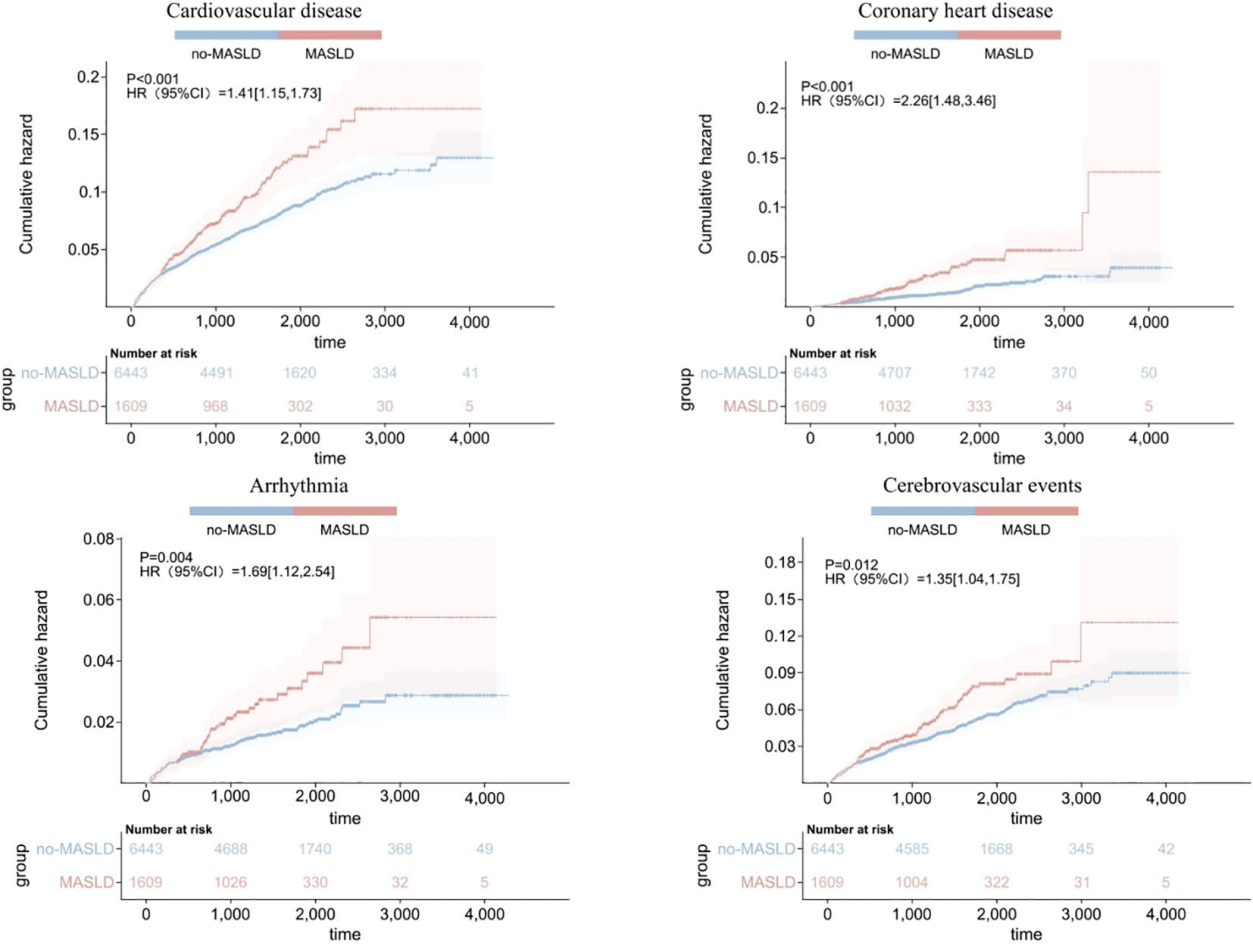
Association between comorbid MASLD and the occurrence of cardiovascular disease.

A total of 594 CVD episodes were found at 32,714 person-years of follow-up, with an incidence density of approximately density 13.37/1000 person-years. MASLD increased overall CVD risk (HR 1.41, 95% CI 1.17-1.70, *p* < 0.001), driven by coronary disease (HR 2.27, 95% CI 1.67-3.21, *p* < 0.001), arrhythmias (HR 1.69, 95% CI 1.18-2.41, *p* = 0.004) and cerebrovascular disease (HR 1.35, 95% CI 1.07-1.72, *p* = 0.013)(**Figure 2**). After adjustment, the risk of coronary disease was 1.91-fold higher in MASLD group (aHR 1.91, 95% CI 1.34-2.74, *p* < 0.001), and persistent risk trends for the others (**Table 2**).

### Subgroup analysis of MASLD associated with clinical outcomes

Stratified analysis by gender revealed a higher incidence of severe liver disease events among male patients compared to the females. Concurrent MASLD significantly reduced the risk of severe liver disease in males (aHR 0.66, 95% CI 0.47-0.94, *p* = 0.021) (**Supplementary Table 1**).

Subgroup analysis by virological characteristics revealed that within the HBsAg-positive stratum, patients with concomitant MASLD exhibited a lower incidence density of severe liver disease. Within this subgroup, those with MASLD demonstrated a significantly reduced risk of severe liver disease (aHR 0.61, 95% CI 0.45-0.84, *p* = 0.002) and hepatocellular carcinoma (aHR 0.59, 95% CI 0.37-0.94, *p* = 0.026) (**Table 3**).

**Table 3:** Stratified subgroup analysis based on HBsAg in the overall cohort.

Clinical outcomes	HBsAg	Total	Cases	Incidence density/per 1000 person-years		aHR	*P* for aHR	*P* for interaction
				noMASLD n=3300	MASLD n=666			
Severe liver disease	–	583	57	0.055	0.061	1.17 (0.60-2.31)	0.648	0.063
	+	3383	429	0.089	0.056	**0.61 (0.45-0.84)**	**0.002**	
HCC	–	583	33	0.028	0.049	2.06 (0.93-4.56)	0.077	**0.006**
	+	3383	219	0.043	0.026	**0.59 (0.37-0.94)**	**0.026**	
Cirrhosis	–	583	21	0.023	0.005	0.21 (0.03-1.60)	0.132	0.400
	+	3383	189	0.037	0.021	**0.55 (0.33-0.92)**	**0.021**	
Cardiovascular disease	–	583	91	0.094	0.091	0.68 (0.39-1.17)	0.165	**0.023**
	+	3383	312	0.051	0.103	**1.42 (1.09-1.86)**	**0.009**	
Coronary heart disease	–	583	29	0.025	0.036	1.09 (0.46-2.61)	0.843	0.136
	+	3383	72	0.010	0.031	**2.25 (1.35-3.73)**	**0.002**	
Arrhythmia	–	583	23	0.022	0.021	0.58 (0.20-1.75)	0.337	0.157
	+	3383	80	0.012	0.030	**1.98 (1.20-3.26)**	**0.007**	
Heart failure	–	583	12	0.012	0.005	0.36 (0.05-2.88)	0.337	0.126
	+	3383	32	0.005	0.011	1.84 (0.82-4.14)	0.142	
Cerebrovascular events	–	583	65	0.061	0.078	0.87 (0.48-1.60)	0.660	0.424
	+	3383	195	0.032	0.056	1.15 (0.81-1.62)	0.436	

Adjusted for age, sex, BMI, hypertension, diabetes and FIB4. *P*-values<0.05 are shown in bold.

Stratified analysis by HBV DNA levels revealed a marginally lower incidence density of severe liver disease among HBV patients with HBV DNA <2000 IU/mL and MASLD. Furthermore, HBV patients with lower HBV DNA levels who developed MASLD exhibited a significantly reduced risk of severe liver disease (aHR 0.59, 95% CI 0.44-0.78, *p* < 0.001), HCC (aHR 0.56, 95% CI 0.36-0.87, *p* = 0.010), and cirrhosis (aHR 0.56, 95% CI 0.37-0.84, *p* = 0.005). Conversely, in the HBV DNA ≥2000 IU/mL cohort, concomitant MASLD significantly promoted cardiovascular disease occurrence in CHB patients (aHR 2.05, 95% CI 1.34-3.13, *p* = 0.001), particularly manifested as significantly increased risks of coronary artery disease (aHR 3.63, 95% CI 1.63-8.09, *p* = 0.002) and cerebrovascular disease (aHR 2.42, 95% CI 1.41-4.15, *p* = 0.001) (**Table 4**).

**Table 4:** Stratified subgroup analysis based on HBV DNA in the overall cohort.

Clinical outcomes	HBsAg	Total	Cases	Incidence density /per 1000 person-years		aHR	*P* for aHR	*P* for interaction
				noMASLD n=5011	MASLD n=1329			
Severe liver disease	<2000	4539	423	0.075	0.045	**0.59 (0.44-0.78)**	**<0.001**	0.362
	≥2000	1801	227	0.101	0.075	0.86 (0.58-1.27)	0.447	
HCC	<2000	4539	189	0.032	0.018	**0.56 (0.36-0.87)**	**0.010**	0.969
	≥2000	1801	95	0.041	0.022	0.61 (0.31-1.20)	0.151	
Cirrhosis	<2000	4539	217	0.037	0.021	**0.56 (0.37-0.84)**	**0.005**	0.143
	≥2000	1801	123	0.051	0.045	1.11 (0.68-1.83)	0.680	
Cardiovascular disease	<2000	4539	334	0.050	0.064	0.99 (0.77-1.28)	0.931	0.123
	≥2000	1801	121	0.042	0.077	**2.05 (1.34-3.13)**	**0.001**	
Coronary heart disease	<2000	4539	29	0.011	0.019	1.50 (0.92-2.44)	0.105	0.059
	≥2000	1801	72	0.007	0.029	**3.63 (1.63-8.09)**	**0.002**	
Arrhythmia	<2000	4539	88	0.012	0.020	1.37 (0.86-2.20)	0.187	0.866
	≥2000	1801	29	0.010	0.018	1.84 (0.77-4.43)	0.172	
Heart failure	<2000	4539	34	0.005	0.004	0.76 (0.31-1.87)	0.548	0.776
	≥2000	1801	16	0.006	0.004	0.71 (0.14-3.56)	0.672	
Cerebrovascular events	<2000	4539	203	0.031	0.033	0.78 (0.55-1.10)	0.161	**0.043**
	≥2000	1801	72	0.024	0.048	**2.42 (1.41-4.15)**	**0.001**	

Adjusted for age, sex, BMI, hypertension, diabetes and FIB4. *P*-values<0.05 are shown in bold.

### The occurrence of MASLD and clinical outcomes in HBsAg-positive patients with varying HBeAg and HBV DNA levels

Interaction analysis indicated that the coexistence of HBsAg status and MASLD exerted a synergistic interaction on HCC development (OR 0.29, 95% CI 0.12, 0.75, *p* = 0.006). When HBsAg-positive patients also had MASLD, the risk of HCC was lower compared to HBsAg-positive patients without MASLD and HBsAg-negative patients with MASLD (**Supplementary Table 2**). In other words, the presence of MASLD significantly reduces the risk of persistent HBsAg positivity, thereby promoting the clearance of HBsAg. Consequently, further stratified analysis within HBsAg-positive patients revealed that the protective effect of MASLD remained statistically significant only when HBV DNA levels were low or HBeAg-negative (**Supplementary Table 3**).

### Sensitive analysis

The two patient cohorts exhibited certain differences in baseline characteristics, lipid profiles, and serum virological levels. Consequently, we conducted a sensitivity analysis, considering the association between MASLD status and BMI, lipid levels, Ultimately, a 1:3 propensity score matching (with a 0.02 threshold) was employed to adjust for age, sex, underlying conditions, virological characteristics, and antiviral treatment status. From 2,973 patients with complete information, 1,775 were selected to form the propensity-score-matched cohort (484 in the MASLD group and 1,291 in the no-MASLD group). This matching strategy achieved adequate covariate balance, rendering the baseline characteristics of the two groups comparable (**Supplementary Table 4**).

In the PSM cohort, patients in the CHB-MASLD group exhibited a lower cumulative incidence of severe liver disease (HR 0.70, 95% CI 0.51-0.94, *p* = 0.02), cirrhosis (HR 0.58, 95% CI 0.36-0.96, *p* = 0.035) and hepatocellular carcinoma (HR 0.71, 95% CI 0.47–1.09, *p* = 0.117). Conversely, cardiovascular disease (HR 1.44, 95% CI 1.10–1.90, *p* = 0.008) was more prevalent, with coronary heart disease (HR 2.21, 95% CI 1.35–3.61, *p* = 0.002) and arrhythmia (HR 1.83, 95% CI 1.09–3.08, *p* = 0.023) were significantly elevated (**Supplementary Table 5**). PSM cohort stratification analysis similarly revealed a low incidence of severe liver disease among HBsAg-positive patients, with MASLD demonstrating statistically significant protective effects, particularly among those with lower viral loads(**Supplementary Tables 6**, **7**). This aligns with previous findings, demonstrating the stability of the protective effect observed in the general population cohort.

## Discussion

This study aimed to evaluate the clinical impact of combined fatty liver disease on the long-term occurrence of severe liver disease and cardiovascular disease in CHB. Within a total cohort of 8,052 CHB patients, we observed that MASLD was associated with a reduced risk of long-term severe liver disease, including hepatocellular carcinoma and cirrhosis, especially in patients with HBsAg positive. While it was simultaneously linked to an increased risk of cardiovascular disease. This effect remained statistically significant after adjusting for confounding factors such as age, gender, BMI, hypertension, and diabetes.

Previous extensive research had evaluated the impact of fatty liver on the development of severe liver disease in CHB patients. Our study, consistent with most prior investigations, found that MASLD confers a certain degree of protective effect. Stratified analysis revealed that MASLD conferred significant and consistent protection against severe liver disease, hepatocellular carcinoma, and cirrhosis in the subgroup of HBsAg-positive patients with low HBV DNA levels or HBeAg-negative status. We hypothesize this protective effect may be associated with the clearance of HBsAg and HBV (Huang et al., 2024b). Although HBV itself is not typically regarded as a cytopathic virus, it can induce hepatocyte damage by triggering an immune response within the hepatocytes infected with HBV. Chronic liver inflammation and inadequate immune-mediated viral clearance are key factors in the progression of cirrhosis and hepatocellular carcinoma. Relevant studies indicate a negative correlation between hepatic steatosis and HBV viral activity, HBeAg, and HBV DNA (Zheng et al., 2021). Combined with the significant multiplicative interaction identified between MASLD and HBsAg in our study, results demonstrated that HBsAg-positive individuals concurrently presenting with MASLD exhibit a markedly reduced risk of severe liver disease compared to those who are HBsAg positive but without MASLD, or those with MASLD but HBsAg negative. We propose that HBsAg status, elevated HBV DNA levels, or high viral activity constitute significant risk factors for severe liver disease, inherently increasing susceptibility in these patient cohorts. Conversely, the presence of hepatic steatosis may, through certain mechanisms, promote HBsAg clearance or suppress HBV replication in hepatitis B patients, thereby partially reducing the incidence of severe liver disease. However, in patients who have achieved surface antigen seroconversion, persistent unresolved MASLD-induced hepatic inflammatory responses may become a driving force for adverse liver outcomes.

Potential mechanisms include steatosis inhibiting HBsAg and HBV DNA secretion by regulating endoplasmic reticulum stress in hepatocytes (Liu et al., 2022). In early stages, MASLD may induce enhanced immune responses through oxidative stress, obesity-associated inflammatory factors activating NLRP3 inflammasomes, and metabolic reprogramming of obese immune cells (Zhao et al., 2025). This immune activation may also exert a certain clearance effect on HBV. A prospective study revealed that adiponectin, a adipokine reducing hepatic steatosis, exhibits significantly elevated levels in patients with higher HBV DNA titres (Wong et al., 2010). A basic research study further demonstrated that in HepG2-B hepatitis B virus-stabilized cells, adiponectin upregulates viral replication, whereas adiponectin knockdown downregulates viral replication (Yoon et al., 2011). Concurrently, saturated fatty acids upregulate Toll-like receptor 4 (TLR4), whose specific ligands inhibit HBV replication (Zhang et al., 2015). Furthermore, increased fatty acid synthase (FAS) receptors on the membranes of fatty liver cells may induce apoptosis in HBV-infected hepatocytes, with viral release into the bloodstream indirectly enhancing opportunities for HBsAg clearance from serum (Feldstein et al., 2003). Conversely, activation of innate immune cells may exacerbate fatty liver progression. Although TLR4 ligands inhibit HBV replication, TLR4 activation itself stimulates Kupffer cells to produce pro-inflammatory cytokines such as TNF-α, IL-1, IL-6, chemokines, and TGF-β. This promotes hepatic fibrosis, leading to progression from fatty liver to cirrhosis and potentially hepatocellular carcinoma (Seki et al., 2007). Moreover, metabolic-related factors may exert certain influences. For instance, PGC-1α, a crucial transcription factor for gluconeogenesis, typically promotes HBV replication by enhancing HBV transcription. However, studies have revealed reduced expression of PGC-1α in MASLD. Consequently, metabolic alterations induced by MASLD impede HBV replication by downregulating PGC-1α expression (Piccinin et al., 2019).

The relationship between metabolic factors and the future risk of severe liver disease in MASLD and CHB patients is complex. A recent Taiwanese study indicates that the cumulative dose of metabolic risk factors increases the risk of liver-related mortality in chronic hepatitis B patients in a dose-dependent manner. However, after propensity score matching, the mortality risk in SLD patients was lower than in non-SLD patients (Huang et al., 2024a). To address this issue, we employed multivariate survival analysis to further investigate the potential role of metabolic product levels. Results indicated that elevated TG, LDL, and HDL levels reduced the incidence risk of severe liver disease, whereas increased GLU levels constituted an independent risk factor for severe liver disease (**Supplementary Table 8**). Multiple studies supported this finding, demonstrating that higher serum cholesterol levels in patients with mild cirrhosis correlate with better liver function preservation and reduced mortality (Kaplan et al., 2019). A Korean population-based study indicated that low serum cholesterol levels, in the absence of statin use, were significantly associated with increased risk of hepatocellular carcinoma (HCC), suggesting dyslipidemia may represent an independent risk factor and preclinical marker for HCC (Cho et al., 2021). Additional research has proposed that poor glycemic control constitutes a risk factor for HCC development following HBsAg seroclearance (Cheuk-Fung Yip et al., 2018).

Some studies also suggested that the presence of MAFLD in CHB patients was associated with an increased risk of liver-related clinical events and mortality, particularly among HBV-infected patients undergoing liver biopsy. The coexistence of fatty liver disease may increase the risk of HCC by 7.3-fold (Chan et al., 2017). A long-term follow-up cohort study across multiple ethnicities in North America and Europe found that 40% of CHB patients with non-alcoholic steatohepatitis (NASH) developed cirrhosis, with superimposed NASH conferring a higher risk of liver-related outcomes such as HCC or death (van Kleef et al., 2021). This disparity may relate to differing diagnostic approaches for fatty liver disease. Wong et al.’s meta-analysis revealed that in biopsy cohorts, CHB-FL patients exhibited approximately 70% higher baseline cirrhosis incidence than CHB-no FL patients. Non-biopsy cohorts showed no significant difference in cirrhosis distribution between groups, suggesting potential selection bias in biopsy-based studies (Wong et al., 2023). While baseline cirrhosis was reported as a significant risk factor for developing hepatocellular carcinoma (Chan et al., 2017). In clinical practice, although liver biopsy offers greater accuracy than non-invasive diagnostics, its invasive nature means it is rarely performed in CHB patients. Consequently, biopsy cohorts may be subject to selection and indication bias, failing to represent the entire population.

MASLD and hepatitis B virus infection may jointly contribute to the development of cardiovascular disease. This study confirmed that MASLD is risk factors for developing cardiovascular disease in CHB patients, with the most significant association observed for coronary heart disease. Extensive research has demonstrated the association between MASLD and heightened cardiovascular disease risk, supported by a growing body of literature (Lee et al., 2024). Numerous pathophysiological alterations associated with fatty liver disease may induce structural, electrophysiological, and autonomic remodeling of the heart, thereby increasing arrhythmogenic substrates within cardiac tissue (Chen et al., 2021). Concurrently, studies indicate that the CVD incidence burden among any liver disease patients, including autoimmune liver disease, viral hepatitis, and fatty liver disease, is twice that of individuals without liver disease. Both hepatitis B and fatty liver disease exhibit higher CVD incidence rates than the non-liver disease population, with coronary heart disease and arrhythmia being the two most common initial manifestations of CVD (Chang et al., 2022). Relevant studies indicated that HBV infection independently promotes atherosclerosis by facilitating subclinical carotid plaque formation (Riveiro-Barciela et al., 2021), correlating with elevated risks of atherosclerosis and coronary heart disease (Wu et al., 2024). Evidence further indicated that MAFLD patients with concomitant viral hepatitis exhibit over double the incidence of cardiovascular disease compared to uninfected individuals (Guerreiro et al., 2021).

Therefore, although MASLD confers a certain protective effect against liver disease progression in CHB patients, the specific cardiovascular hazards associated with MASLD cannot be overlooked. Equally important is that some individuals with CHB who have achieved hepatitis B surface antigen seroconversion combined with MASLD may become a risk factor for severe liver disease. In the clinical management of such patients, it is essential to carefully assess the risk of worsening liver disease while actively pursuing antiviral treatment and implementing proactive measures to prevent the onset of cardiovascular disease. Instead, our findings emphasize the importance of investigating the mechanisms of protective effects of MASLD, as this may provide an opportunity to identify potential therapeutic targets for CHB cure in the future.

Our study also has certain limitations. Firstly, as a single-center retrospective cohort study, the patient population exhibited relatively homogeneous characteristics. Although the sample size was substantial, some data were missing, potentially introducing bias into the results. Secondly, the study did not incorporate specific antiviral treatment regimens or lipid-lowering medication usage information. Furthermore, serum metabolic markers inherently exhibit daily fluctuations. Future research could introduce longitudinal dynamic monitoring of metabolic indicators to further elucidate the mediating role of metabolic levels in the progression from MASLD to severe liver disease. Finally, due to limitations in clinical research data, this study did not conduct further stratified analyses based on MASLD severity or the antigen-antibody status of hepatitis B infected patients. Future studies incorporating such refinements may deepen our understanding of the interactive effects of MASLD and HBV infection on patient clinical outcomes, thereby enhancing the clinical guidance value of this research.

## Data Availability

The raw data supporting the conclusions of this article will be made available by the authors, without undue reservation.

## Supplementary material

Supplementary Figure 1 Flow chart.

## Glossary

MASLD: Metabolic dysfunction-associated steatotic liver disease
CVD: Cardiovascular disease
CHB: Chronic Hepatitis B
FL: Fatty liver
HCC: Hepatocellular carcinoma
HBV: Hepatitis B virus
DM: Diabetes mellitus
CT: Computed tomography
CDSD: The clinical disease-specific database of Jiangsu province hospital
BMI: Body mass index
GLU: Fasting glucose
TC: Total cholesterol
HDL: High-density lipoprotein cholesterol
LDL: Low-density lipoprotein cholesterol
TG: Triglycerides
ALT: Alanine aminotransferase
AST: Aspartate aminotransferase
PLT: Platelet count
FIB-4: Fibrosis-4 Index
HBsAg: Hepatitis B surface antigen
HBsAb: Hepatitis B surface antibody
HBeAg: Hepatitis B e antigen
HBeAb: Hepatitis B e antibody
HBcAb: Hepatitis B c antibody
HBV DNA: Hepatitis B virus deoxyribonucleic acid
SD: Standard deviation
aHR: Adjusted hazard ratio
CI: Confidence interval
NAs: Nucleos(t)ide analogues
NAFLD: Non-alcoholic fatty liver disease
MAFLD: Metabolic-associated fatty Liver disease
NASH: Non-Alcoholic steatohepatitis
MASH: Metabolic dysfunction-associated steatohepatitis
TLR4: Toll-like receptor 4
IHD: Ischemic heart disease
ETV: Entecavir
TDF: Tenofovir disoproxil fumarate
RERI: Relative Excess Risk due to Interaction
AP: Attributable Proportion due to Interaction
SI: Synergy Index
