# Correction: Metabolic dysfunction-associated steatotic liver disease in chronic hepatitis B patients: risks of severe liver disease and cardiovascular disease

**DOI:** 10.3389/fcimb.2026.1907033

**Published:** 2026-06-19

**Authors:** Ningxin Zhang, Zhongwei Xu, Fei Lin, Haozhi Fan, Dehong Zhou, Shiqiu Zheng, Peng Jin, Huiquan Gu, Chengxiao Yu, Longfeng Jiang

**Affiliations:** 1 Department of Infectious Diseases, The First Affiliated Hospital with Nanjing Medical University, Nanjing, Jiangsu, China; 2 Health Management Center, The First Affiliated Hospital with Nanjing Medical University, Nanjing, Jiangsu, China; 3 Department of Information Management, The First Affiliated Hospital with Nanjing Medical University, Nanjing, China; 4 Jiangsu Clinical Medicine Research Institute, Nanjing, China; 5 Department of Health Management, School of Public Health, Nanjing Medical University, Nanjing, Jiangsu, China

**Keywords:** cirrhosis, coronary heart disease, HBsAg, hepatocellular carcinoma, severe liver disease

In the published article, there was a mistake in the **Funding** statement. The funding number for the Basic Research Program of Jiangsu was erroneously displayed as “SBK20250203138”. The correct statement is “Basic Research Program of Jiangsu (BK20251946)”.

The original version of this article has been updated.

