# Supplementary material for: Metabolic dysfunction-associated steatotic liver disease in chronic hepatitis B patients: risks of severe liver disease and cardiovascular disease
Source: Front Cell Infect Microbiol. 2026 Jun 9;16:1806927. doi: 10.3389/fcimb.2026.1806927 (PMC13329344; doi:10.3389/fcimb.2026.1806927)
Supplement: Supplementary Figure 1 — Flow chart. [file DataSheet1.docx]

**Supplementary material**

Figure S1. flow chart.


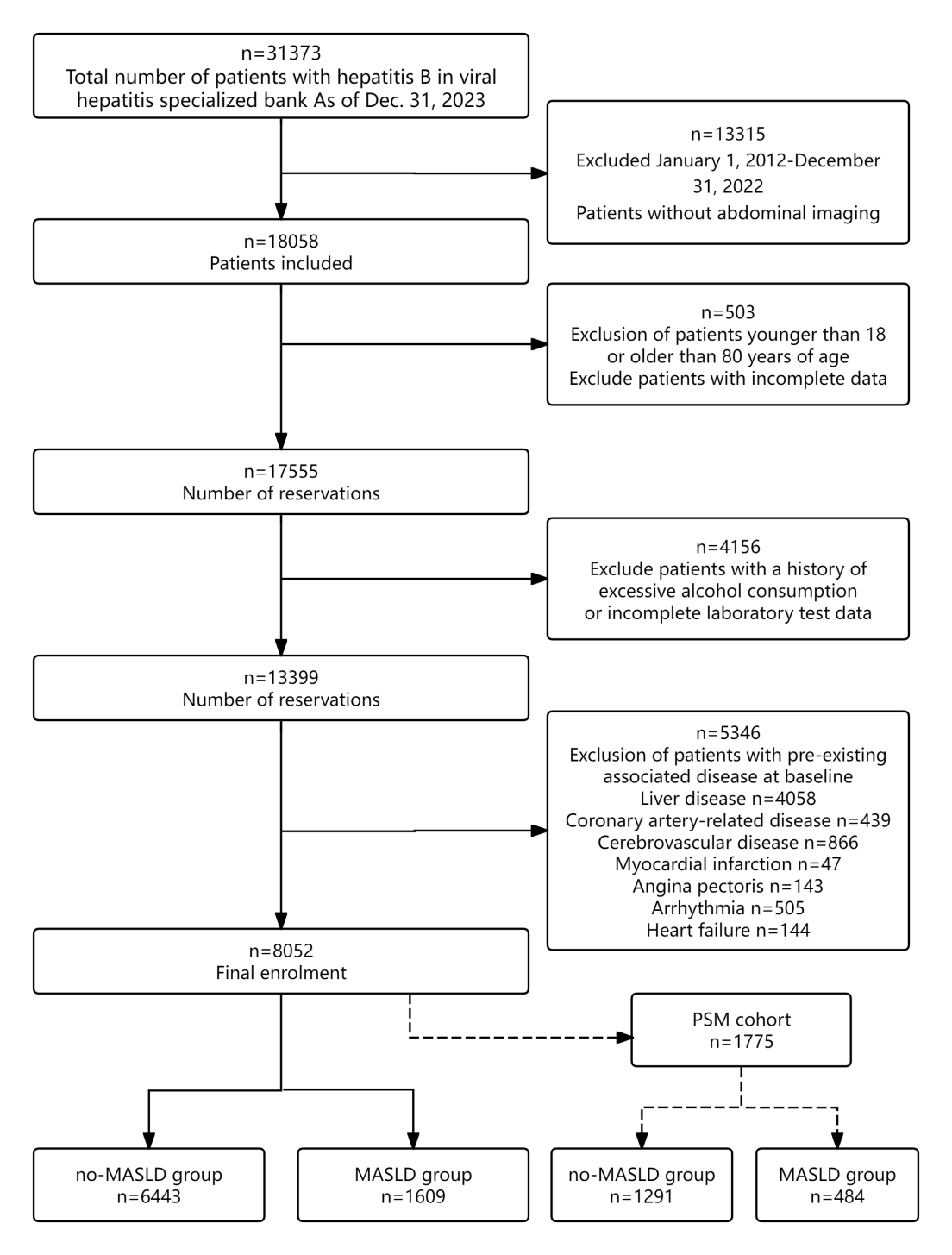


Table S1. Stratified subgroup analysis based on sex in the overall cohort.

| **Clincal outcomes** | **Sex** | **Total** | **cases** | **No-MASLD (n=2469)** | **MASLD (n=504)** | **aHR** | ***P*** |
| --- | --- | --- | --- | --- | --- | --- | --- |
| Severe liver disease | female | 1271 | 93 | 0.049 | 0.048 | 0.79(0.42-1.47) | 0.452 |
|  | male | 1702 | 316 | 0.140 | 0.087 | **0.66(0.47-0.94)** | **0.021** |
| HCC | female | 1271 | 40 | 0.021 | 0.016 | 0.52(0.18-1.50) | 0.227 |
|  | male | 1702 | 170 | 0.069 | 0.048 | 0.76(0.48-1.21) | 0.250 |
| Cirrhosis | female | 1271 | 48 | 0.024 | 0.028 | 0.99(0.43-2.24) | 0.974 |
|  | male | 1702 | 131 | 0.055 | 0.024 | **0.47(0.25-0.89)** | **0.019** |
| Cardiovascular disease | female | 1271 | 120 | 0.053 | 0.147 | **1.69(1.12-2.55)** | **0.012** |
|  | male | 1702 | 191 | 0.069 | 0.090 | 1.02(0.71-1.47) | 0.912 |
| Coronary heart disease | female | 1271 | 28 | 0.010 | 0.040 | 1.79(0.80-4.01) | 0.154 |
|  | male | 1702 | 49 | 0.014 | 0.034 | **1.81(0.95-3.46)** | **0.071** |
| Arrhythmia | female | 1271 | 36 | 0.015 | 0.040 | 1.81(0.85-3.84) | 0.121 |
|  | male | 1702 | 46 | 0.015 | 0.026 | 1.30(0.64-2.66) | 0.470 |
| Heart failure | female | 1271 | 8 | 0.003 | 0.012 | 3.48(0.77-15.71) | 0.105 |
|  | male | 1702 | 30 | 0.011 | 0.010 | 0.84(0.30-2.32) | 0.731 |
| Cerebrovascular events | female | 1271 | 75 | 0.032 | 0.088 | 1.53(0.91-2.57) | 0.107 |
|  | male | 1702 | 118 | 0.043 | 0.048 | 0.86(0.53-1.40) | 0.539 |

Adjusted for age, BMI, hypertension, diabetes, HBsAg, HBeAg, log HBV DNA and FIB4.

Table S2. Analysis of the Interaction Between HBsAg Positivity and MASLD on the Risk of HCC Development.

|  | HBsAg - | HBsAg + | Effect of HBsAg within the strata of MASLD |
| --- | --- | --- | --- |
|  | OR [95% CI] | OR [95% CI] | OR [95% CI] |
| MASLD absent | 1 [Reference] | 1.53 [0.98, 2.4] | 1.53 [0.98, 2.4] |
| MASLD present | 1.77 [0.79, 3.99] | 0.8 [0.43, 1.47] | 0.45 [0.2, 1.02] |
| Effect of MASLD within the strata of HBsAg | 1.77 [0.79, 3.99] | 0.52 [0.33, 0.83] |  |
| Multiplicative scale | **0.29 [0.12, 0.75]** |  |  |
| RERI | -1.5 [-4.09, -0.36] |  |  |
| AP | -1.89 [-5.18, -0.46] |  |  |
| SI | - |  |  |

RERI Relative excess hazard attributable to interaction, AP Attribution ratio of interactions, SI Collaboration Index. Adjusted for age, sex, hypertension, diabetes, BMI and FIB4.

Table S3. Stratified subgroup analysis in the HBsAg positive cohort.

| **Clincal outcomes** | **group** | **Total** | **cases** | **noMASLD** | **MASLD** | **aHR** | ***P*** |
| --- | --- | --- | --- | --- | --- | --- | --- |
| **grouped by HBV DNA** |  |  |  | **n=1294** | **n=450** |  |  |
| Severe liver disease | ＜2000 | 1763 | 233 | 0.097 | 0.066 | **0.63(0.42-0.95)** | **0.026** |
|  | ≥2000 | 881 | 141 | 0.120 | 0.082 | 0.73(0.42-1.27) | 0.268 |
| HCC | ＜2000 | 1809 | 123 | 0.049 | 0.032 | **0.61(0.34-1.07)** | **0.085** |
|  | ≥2000 | 826 | 64 | 0.050 | 0.031 | 0.64(0.21-1.53) | 0.317 |
| Cirrhosis | ＜2000 | 1809 | 99 | 0.039 | 0.023 | **0.56(0.29-1.09)** | **0.090** |
|  | ≥2000 | 826 | 69 | 0.054 | 0.036 | 0.75(0.34-1.68) | 0.489 |
| **grouped by HBeAg** |  |  |  | **n=2813** | **n=551** |  |  |
| Severe liver disease | - | 2722 | 345 | 0.090 | 0.054 | **0.56(0.39-0.81)** | **0.002** |
|  | + | 642 | 81 | 0.085 | 0.071 | 0.75(0.37-1.51) | 0.413 |
| HCC | - | 2722 | 181 | 0.045 | 0.027 | **0.57(0.35-0.95)** | **0.031** |
|  | + | 642 | 38 | 0.039 | 0.022 | 0.53(0.16-1.75) | 0.299 |
| Cirrhosis | - | 2722 | 148 | 0.037 | 0.019 | **0.50(0.28-0.90)** | **0.020** |
|  | + | 642 | 38 | 0.038 | 0.030 | 0.74(0.26-2.13) | 0.577 |

Adjusted for age, sex, BMI, hypertension, diabetes and FIB4.

Table S4. Baseline clinical characteristics after propensity score matching.

|  | **Before PSM** | | | | **After PSM** | | | | **test**  **SMD** |
| --- | --- | --- | --- | --- | --- | --- | --- | --- | --- |
|  | **Overall (**n=2973**)** | **CHB- no MASLD (**n=2469) | **CHB- MASLD (**n=504**)** | ***p*** | **Overall**  **(**n=1775**)** | **CHB-no MASLD (**n=1291) | **CHB- MASLD (**n=484**)** | ***p*** |  |
| age | 49.56±12.55 | 49.78±12.77 | 48.52 ±11.37 | **0.040** | 49.16±12.60 | 49.31±13.03 | 48.75±11.40 | 0.405 | **0.046** |
| Male sex  (n, %) | 1702 (57.2) | 1372 (55.6) | 330 (65.5) | **<0.001** | 1115 (62.8) | 805 (62.4) | 310 (64.0) | 0.547 | **0.035** |
| hypertension | 857 (28.8) | 642 (26.0) | 215 (42.7) | **<0.001** | 661 (37.2) | 466 (36.1) | 195 (40.3) | 0.116 | **0.086** |
| diabetes | 526 (17.7) | 395 (16.0) | 131(26.0) | **<0.001** | 395 (22.3) | 279 (21.6) | 116 (24.0) | 0.318 | **0.056** |
| HBsAg positive | 2650 (89.1) | 2199 (89.1) | 451 (89.5) | **0.844** | 1575 (88.7) | 1144 (88.6) | 431 (89.0) | 0.861 | **0.014** |
| HBeAg positive | 553 (18.6) | 465 (18.8) | 88 (17.5) | **0.510** | 311 (17.5) | 224 (17.4) | 87 (18.0) | 0.812 | **0.016** |
| Log HBV DNA | 3.06±1.73 | 3.08 ±1.73 | 2.94 ±1.70 | **0.077** | 2.99 ±1.72 | 3.01±1.72 | 2.96±1.72 | 0.608 | **0.027** |
| Anti Virus | 1328 (44.7) | 1126 (45.6) | 202 (40.1) | **0.026** | 772 (43.5) | 573 (44.4) | 199 (41.1) | 0.237 | **0.066** |

Table S5. The relationship between MASLD and clinical outcomes in PSM cohort.

| **Characteristic** | **No. of participants** | **No. of cases** | **Uni-variation** | | **Multi-variation** | |
| --- | --- | --- | --- | --- | --- | --- |
|  |  |  | **HR(95.0%CI)** | ***P*** | **HR(95.0%CI)** | ***P*** |
| Severe liver disease | 1775 | 229 | **0.70(0.51-0.97)** | **0.031** | **0.74(0.54-1.04)** | **0.085** |
| HCC | 1775 | 114 | 0.81(0.52-1.25) | 0.331 | 0.89(0.57-1.40) | 0.611 |
| Cirrhosis | 1775 | 101 | **0.53(0.31-0.90)** | **0.024** | **0.57(0.33-0.98)** | **0.004** |
| Cardiovascular disease | 1775 | 216 | 1.24(0.92-1.66) | 0.157 | 1.20(0.89-1.61) | 0.237 |
| Coronary heart disease | 1775 | 58 | **2.38(1.41-4.00)** | **0.001** | **2.01(1.17-3.44)** | **0.012** |
| Arrhythmia | 1775 | 56 | **1.65(0.95-2.85)** | **0.073** | 1.36(0.78-2.38) | 0.280 |
| Heart failure | 1775 | 23 | 1.08(0.42-2.73) | 0.878 | 1.07(0.41-2.80) | 0.897 |
| Cerebrovascular events | 1775 | 136 | 1.09(0.75-1.59) | 0.653 | 1.10(0.75-1.61) | 0.636 |

Multi-variation was adjusted for sex, age, BMI, hypertension, diabetes, log HBV DNA and FIB4.

Table S6. Stratified subgroup analysis based on sex in the PSM cohort.

| **Clincal outcomes** | **HBsAg** | **Total** | **cases** | **incidence density /per 1000 personyears** | | **aHR** | ***P*for  aHR** |
| --- | --- | --- | --- | --- | --- | --- | --- |
|  |  |  |  | **noMASLD** **n=3300** | **MASLD** **n=666** |  |  |
| Severe liver disease | - | 200 | 23 | 0.067 | 0.090 | 1.17(0.47-2.91) | 0.740 |
|  | + | 1575 | 206 | 0.100 | 0.069 | **0.68(0.47-0.98)** | **0.038** |
| HCC | - | 200 | 17 | 0.045 | 0.076 | 1.57(0.56-4.44) | 0.391 |
|  | + | 1575 | 97 | 0.043 | 0.033 | 0.78(0.46-1.33) | 0.359 |
| Cirrhosis | - | 200 | 6 | 0.020 | 0.012 | 0.57(0.06-5.19) | 0.615 |
|  | + | 1575 | 95 | 0.045 | 0.025 | **0.56(0.31-1.00)** | **0.049** |
| Cardiovascular disease | - | 200 | 34 | 0.129 | 0.063 | 0.44(0.16-1.21) | 0.110 |
|  | + | 1575 | 182 | 0.071 | 0.105 | 1.39(1.01-1.91) | 0.047 |
| Coronary heart disease | - | 200 | 11 | 0.031 | 0.036 | 0.78(0.16-3.81) | 0.754 |
|  | + | 1575 | 47 | 0.013 | 0.036 | **2.46(1.33-4.53)** | **0.004** |
| Arrhythmia | - | 200 | 11 | 0.040 | 0.012 | 0.18(0.02-1.58) | 0.120 |
|  | + | 1575 | 45 | 0.014 | 0.031 | **1.87(1.01-3.46)** | **0.047** |
| Heart failure | - | 200 | 5 | 0.020 | 0 | - | - |
|  | + | 1575 | 18 | 0.006 | 0.010 | 1.56(0.55-4.47) | 0.406 |
| Cerebrovascular events | - | 200 | 22 | 0.071 | 0.063 | 0.89(0.31-2.58) | 0.824 |
|  | + | 1575 | 114 | 0.046 | 0.054 | 1.14(0.75-1.74) | 0.538 |

Adjusted for age, sex, BMI, hypertension, diabetes, HBeAg, GLU, TG, FIB4 and HDL＜1.

Table S7. Stratified subgroup analysis based on HBV DNA in the PSM cohort with HBsAg positive.

| **Clincal outcomes** | **group** | **Total** | **cases** | **noMASLD** | **MASLD** | **aHR** | ***P*** |
| --- | --- | --- | --- | --- | --- | --- | --- |
| **grouped by HBV DNA** |  |  |  | **n=1144** | **n=431** |  |  |
| Severe liver disease | ＜2000 | 1073 | 133 | 0.095 | 0.063 | **0.68(0.44-1.06)** | **0.091** |
|  | ≥2000 | 489 | 72 | 0.111 | 0.084 | 0.77(0.43-1.40) | 0.395 |
| HCC | ＜2000 | 1073 | 65 | 0.042 | 0.034 | 0.80(0.44-1.47) | 0.474 |
|  | ≥2000 | 489 | 32 | 0.047 | 0.031 | 0.76(0.30-1.90) | 0.557 |
| Cirrhosis | ＜2000 | 1073 | 59 | 0.042 | 0.019 | **0.50(0.23-1.06)** | **0.069** |
|  | ≥2000 | 489 | 35 | 0.051 | 0.037 | 0.76(0.32-1.79) | 0.528 |
| **grouped by HBeAg** |  |  |  | **n=1144** | **n=431** |  |  |
| Severe liver disease | - | 1273 | 168 | 0.102 | 0.065 | **0.63(0.42-0.95)** | **0.026** |
|  | + | 302 | 38 | 0.090 | 0.088 | 0.84(0.38-1.84) | 0.657 |
| HCC | - | 1273 | 82 | 0.045 | 0.034 | 0.71(0.41-1.24) | 0.232 |
|  | + | 302 | 15 | 0.035 | 0.027 | 0.82(0.22-3.12) | 0.772 |
| Cirrhosis | - | 1273 | 76 | 0.045 | 0.022 | **0.53(0.28-1.03)** | **0.060** |
|  | + | 302 | 19 | 0.044 | 0.037 | 0.67(0.21-2.13) | 0.492 |

Adjusted for age, sex, BMI, hypertension, diabetes and FIB4.

Table S8. Potential risk factors associated with the occurrence of severe liver disease

| **Variables** | **aHR** | **95%CI** | **P** |
| --- | --- | --- | --- |
| MASLD | 0.78 | 0.62-0.97 | 0.025 |
| age | 1.03 | 1.02-1.04 | <0.001 |
| sex | 1.87 | 1.57-2.24 | <0.001 |
| BMI | 0.99 | 0.97-1.01 | 0.500 |
| FIB4 | 1.02 | 1.01-1.02 | <0.001 |
| Glu | 1.04 | 1.03-1.05 | <0.001 |
| TG | 0.65 | 0.56-0.75 | <0.001 |
| TC | 1.06 | 0.88-1.28 | 0.564 |
| LDL | 0.74 | 0.59-0.94 | 0.013 |
| HDL | 0.72 | 0.54-0.97 | 0.031 |
| Hypertension | 1.12 | 0.95-1.32 | 0.162 |

BMI body mass index, FIB-4 fibrosis-4 Index, GLU fasting glucose, TG triglycerides，TC total cholesterol, LDL low-density lipoprotein cholesterol, HDL high-density lipoprotein cholesterol .
